# Vertebral trabecular attenuation serial changes on chest CT after autologous hematopoietic stem cell transplantation

**DOI:** 10.1007/s00256-026-05316-x

**Published:** 2026-07-27

**Authors:** Zeynep Atçeken, Sezer Kula, Taner Tan, Umur Topcu, Çetin Atasoy, Yüksel Peker

**Affiliations:** 1 Department of Radiology, Koc University School of Medicine, Istanbul, Türkiye; 2 Department of Hematology, Yalova Training and Research Hospital, Yalova, Türkiye; 3 Department of Emergency Medicine, Zonguldak Alaplı State Hospital, Zonguldak, Türkiye; 4 Department of Pulmonary Medicine, Koc University School of Medicine, Istanbul, Türkiye; 5 Department of Molecular and Clinical Medicine, Institute of Medicine, Sahlgrenska Academy, University of Gothenburg, Gothenburg, Sweden; 6 Department of Clinical Sciences, Respiratory Medicine and Allergology, Faculty of Medicine, Lund University, Lund, Sweden; 7 Division of Pulmonary, Allergy, and Critical Care Medicine, University of Pittsburgh School of Medicine, Pittsburgh, PA USA

**Keywords:** Autologous hematopoietic stem cell transplantation, Vertebral trabecular attenuation, Osteoporosis, Chest CT

## Abstract

**Objective:**

Transplant-associated bone disease and fragility fractures are recognized complications after hematopoietic stem cell transplantation (HSCT). We aimed to evaluate early and longitudinal changes in vertebral trabecular attenuation on routine chest CT in patients undergoing autologous HSCT.

**Materials and methods:**

In this retrospective single-centre study, 98 adult patients who underwent autologous HSCT and had both pre-transplant and early post-transplant (≤ 30 days) non-contrast chest CT were included. Vertebral trabecular attenuation was measured in Hounsfield units (HU) at T10–T12 and vertebral body height was measured in millimeters. Longitudinal changes were additionally evaluated in patients with available late follow-up CT (> 30 days) (*n* = 61).

**Results:**

Vertebral trabecular attenuation decreased significantly from pre-transplant to early post-transplant CT (163.0 ± 57.0 HU vs. 132.7 ± 53.9 HU; *p* < 0.001). In patients with late follow-up imaging, attenuation remained lower than baseline (157.3 ± 58.7 HU vs. 125.3 ± 56.1 HU; *p* < 0.001). The proportion of attenuation values within the osteoporosis range (≤ 110 HU) increased from 20.4% at baseline to 39.8% early after transplantation. Among patients with late follow-up imaging, this proportion increased from 27.4% to 45.2% early after transplantation and remained elevated at 48.4% at late follow-up. Vertebral body height remained unchanged during the early post-transplant period but decreased significantly at late follow-up.

**Conclusion:**

Vertebral trabecular attenuation on routine chest CT declines within the first month after autologous HSCT and remains reduced on follow-up imaging. Opportunistic CT-based assessment may enable early identification of skeletal vulnerability.

## Introduction

Transplant-associated bone disease represents an important source of morbidity in patients undergoing hematopoietic stem cell and solid organ transplantation. Increased risks of osteoporosis and fragility fractures have been consistently reported across transplant populations compared with the general population. Population-based studies have demonstrated a substantially elevated risk of osteoporosis and fractures after organ transplantation [1]. In lung transplant recipients, fractures have been reported as a major clinical problem within the first post-transplant year despite preventive strategies [2]. Previous studies have demonstrated substantially increased risks of osteoporosis and fragility fractures following transplantation, with fracture incidence reported to be particularly high during the first post-transplant year despite preventive strategies. These findings highlight the clinical importance of early skeletal vulnerability and the need for timely bone health assessment in transplant recipients.

The pathogenesis of post-transplant bone loss is multifactorial. Glucocorticoid exposure, immunosuppressive therapy, reduced physical activity, endocrine disturbances, nutritional deficiencies, and persistent metabolic abnormalities have all been implicated in transplant-related bone disease [3, 4]. In addition, alterations in mineral metabolism may persist even after transplantation, contributing to ongoing skeletal fragility [5]. These mechanisms collectively contribute to accelerated bone loss and increased fracture risk during the post-transplant period.

Despite recognition of early bone loss after transplantation, skeletal evaluation in clinical practice typically relies on dual-energy X-ray absorptiometry (DXA), which is often performed months after transplantation [6]. As a result, potential skeletal changes occurring during the immediate peri-transplant period may remain undetected. Earlier identification of skeletal vulnerability could enable timely preventive strategies and improve bone health management in transplant recipients.

In parallel, opportunistic assessment of bone attenuation using routine CT has gained increasing attention. Vertebral trabecular attenuation measured in Hounsfield units (HU) on routine CT has been shown to correlate with bone mineral density and fracture risk. Large population-based studies have established reference values for vertebral attenuation, supporting the feasibility of CT-based opportunistic osteoporosis screening without additional imaging or radiation exposure [7]. Furthermore, vertebral CT attenuation has been reported to outperform traditional fracture risk prediction tools in detecting osteoporosis in lung cancer screening populations [8]. In transplant candidates, CT-derived vertebral attenuation measurements have also demonstrated diagnostic value for opportunistic osteoporosis screening [9].

Patients undergoing autologous hematopoietic stem cell transplantation (HSCT) frequently undergo chest CT during the peri-transplant period for clinical indications [10]. However, pre- to early post-transplant changes in vertebral trabecular attenuation—particularly within the first month after transplantation—have not been systematically evaluated. Detecting attenuation decline during this early period could provide an opportunity for early identification of skeletal vulnerability using imaging that is already routinely performed.

The aim of this study was therefore to evaluate early and longitudinal changes in vertebral trabecular attenuation measured on routine chest CT in adults undergoing autologous HSCT and to explore the potential role of opportunistic CT-based assessment for early detection of skeletal risk.

## Materials and methods

### Study design and population

This retrospective observational single-centre study was approved by the Institutional Review Board (IRB) (approval number: 2026.110.IRB.050). The study was conducted in accordance with institutional and international ethical standards. The requirement for informed consent was waived due to the retrospective nature of the study.

Chest CT examinations of 460 adult patients who underwent autologous hematopoietic stem cell transplantation (HSCT) between 11 January 2022 and 15 December 2025 were retrospectively reviewed. Pre-transplant chest CT examinations were routinely performed as part of standard pre-transplant evaluation to exclude pulmonary pathology. Post-transplant CT examinations were obtained according to clinical indications rather than as part of a routine imaging protocol. The clinical indication for each early post-transplant CT examination was retrospectively retrieved from CT request forms and electronic medical records and categorized according to the primary documented reason for imaging as follows: fever and/or suspected infection, respiratory symptoms and/or hypoxemia, or unexplained clinical deterioration.

For analytical purposes, post-transplant CT examinations were categorized as early post-transplant CT, ≤ 30 days after transplantation, and late follow-up CT, > 30 days after transplantation.

Patients were included if they were *1)* ≥ 18 years of age, 2*)* had both pre- and post-transplant non- enhanced chest CT examinations, *3)* had scans acquired at 120 kVp.

Patients were excluded if they lacked early post-transplant CT, had contrast-enhanced CT examinations, or had CT acquisitions with inconsistent technical parameters, in order to minimize attenuation variability related to intravenous contrast administration and acquisition differences. Additional exclusion criteria were limited to vertebral conditions involving the measured vertebral levels (T10–T12) that could affect attenuation or vertebral height measurements, including vertebral metastasis, pre-existing vertebral compression fracture, severe deformity, focal sclerosis, or prior spinal instrumentation. Vertebral abnormalities outside the measured T10–T12 levels were not considered exclusion criteria unless they affected image quality or prevented standardized measurement at the evaluated levels.

**Fig. 1:**
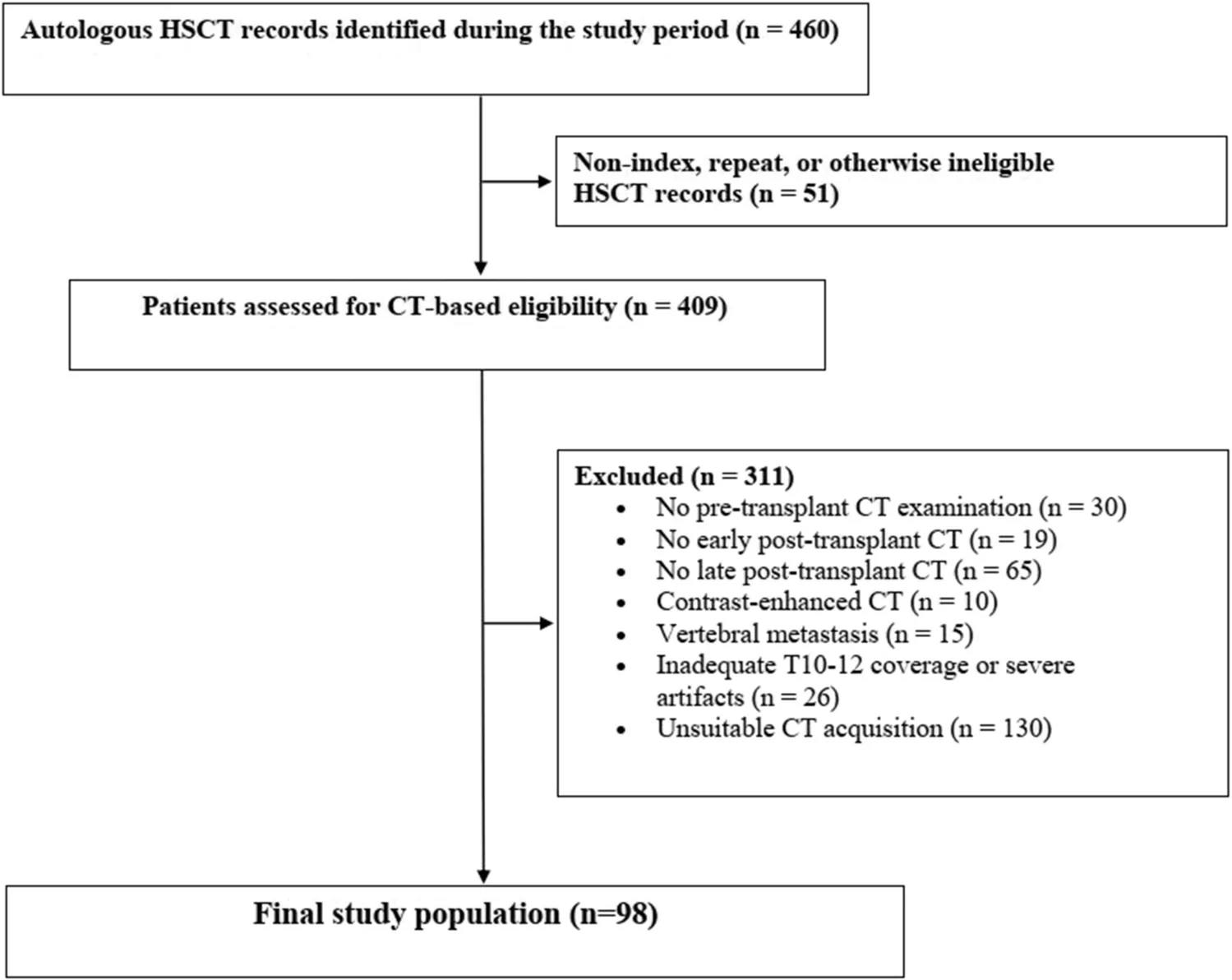
Flowchart of patient selection and exclusion criteria. A total of 460 autologous HSCT patients with available chest CT examinations were screened. After applying the predefined inclusion and exclusion criteria, 98 patients were included in the final analysis

After application of these criteria, 98 patients constituted the final study cohort (Fig. 1).

For analytic purposes, the study population was divided into 2 overlapping cohorts. The first cohort consisted of all patients with available pre-transplant and early post-transplant CT examinations and was used to evaluate immediate attenuation changes following transplantation. The second cohort consisted of patients with available late follow-up CT imaging (> 30 days after transplantation). All patients included in the late follow-up cohort had also undergone early post-transplant CT examinations. This subgroup was analyzed separately to assess longitudinal attenuation changes over time.

Demographic and clinical data including age, sex, and underlying diagnosis were retrieved from electronic medical records. All patients underwent autologous HSCT. Patients with lymphoma received BEAM (carmustine, etoposide, cytarabine, and melphalan) conditioning, whereas patients with multiple myeloma received melphalan-based conditioning regimens. No patient received total body irradiation or post-transplant immunosuppressive therapy. Exposure to glucocorticoids was limited to standardized premedication administered as part of the conditioning regimen according to institutional protocols.

### CT acquisition protocol

All chest CT examinations were performed using 64-detector multidetector CT scanners (Somatom Definition AS and Somatom Definition Flash; Siemens Healthineers, Forchheim, Germany). Identical acquisition protocols were applied across scanners.

Scans were obtained without intravenous contrast administration. Patients were scanned in the supine position during end-inspiratory breath-hold with arms elevated above the head. The scanning range extended from the lung apices to the costophrenic angles.

Tube current was automatically modulated according to patient body habitus using automatic exposure control. Images were reconstructed using a medium soft-tissue kernel (e.g., B30f or equivalent) with slice thickness of 1 mm and a reconstruction increment of 1 mm. Standardised acquisition parameters were used to minimise inter-scan variability. This approach ensured consistency and reproducibility of attenuation measurements.

### Vertebral trabecular attenuation measurement

Vertebral trabecular attenuation was measured in Hounsfield units (HU). Measurements were obtained from the vertebral bodies of T10, T11, and T12. Vertebral levels were identified on sagittal reconstructions, and attenuation measurements were performed on axial images at the mid-vertebral level to ensure accurate and reproducible assessment of trabecular bone.

For each vertebral body, a circular region of interest (ROI) of approximately 1 cm^2^ was placed in the central trabecular portion of the vertebral body on axial images at the mid-vertebral level. Care was taken to avoid cortical bone, endplates, venous plexus structures, focal sclerosis, and visible lesions.

The mean attenuation value of T10–T12 vertebral bodies was calculated and used as the representative vertebral trabecular attenuation for each examination. T10–T12 vertebral bodies were selected because they were consistently included on chest CT examinations and allowed standardized attenuation assessment across patients and time points.

Attenuation change was calculated as: ΔHU = post-transplant HU − pre-transplant HU.

To assess intraobserver reproducibility, vertebral attenuation measurements were repeated by the same senior radiologist at a separate session one month later using the same measurement method.

### Vertebral body height measurement

Vertebral body height was measured at T10, T11, and T12 using sagittal reformatted CT images. Measurements were performed on the mid-sagittal plane of each vertebral body. Anterior vertebral height was defined as the maximum craniocaudal distance between the superior and inferior endplates at the anterior third of the vertebral body. Middle vertebral height was measured at the midpoint of the vertebral body, and posterior vertebral height was defined as the maximum craniocaudal distance between the superior and inferior endplates at the posterior third of the vertebral body. Care was taken to align the calipers perpendicular to the endplates and to avoid osteophytes or endplate irregularities.

To assess measurement reproducibility, vertebral body height measurements were repeated by the same senior radiologist during a separate session, and the mean of the two measurements was used for the primary analyses. In addition, vertebral body height measurements were independently performed by a second radiologist using the same measurement protocol. For both readers, CT examinations were evaluated in a blinded manner, with patient identifiers, scan dates, clinical information, and the chronological order of the examinations concealed during the measurement process. Interobserver agreement was assessed using the intraclass correlation coefficient (ICC).

### Exploratory threshold-based analysis

To explore clinically relevant attenuation categories, vertebral trabecular attenuation values were evaluated using previously reported CT-based reference thresholds. Prior studies have suggested that attenuation values ≤ 110 HU on routine CT may be associated with low bone mineral density and increased likelihood of osteoporosis [11].

### Statistical analysis

Statistical analyses were performed using IBM SPSS Statistics for Windows, Version 28.0 (IBM Corp., Armonk, NY, USA). Continuous variables are presented as mean ± standard deviation (SD) or median with interquartile range (IQR), depending on data distribution. Normality of paired differences was assessed using the Shapiro–Wilk test.

Changes between pre-transplant and early post-transplant vertebral attenuation as well as vertebral body heigth measurement values were evaluated using the Wilcoxon signed-rank test. In patients with available late follow-up CT, longitudinal changes between time points were assessed using either the Wilcoxon signed-rank test or paired t-test, depending on normality.

To explore factors associated with early attenuation decline, linear regression analysis was performed with change in vertebral attenuation between pre-transplant and early post-transplant CT (ΔHU) as the dependent variable. Independent variables included age, sex, and baseline vertebral attenuation.

The proportion of patients with attenuation values within the osteoporosis range (≤ 110 HU) was calculated for each time point. Transition analysis was performed to evaluate changes in osteoporosis-range attenuation (≤ 110 HU) between time points.

Intraobserver agreement for vertebral trabecular attenuation measurements was assessed using the intraclass correlation coefficient (ICC) based on repeated measurements performed by the same observer one month apart. Interobserver agreement for vertebral body height measurements was assessed using ICC based on independent measurements performed by a second radiologist who was blinded to the initial measurements and clinical information. ICC values were calculated separately for anterior, middle, and posterior vertebral body height measurements at the pre-transplant, early post-transplant, and late follow-up CT examinations.

A *p* value < 0.05 was considered statistically significant.

## Results

### Patient characteristics

A total of 460 autologous HSCT records were identified during the study period. After removal of non-index, repeat, or otherwise ineligible HSCT records, 409 patients were assessed for CT-based eligibility. After application of the predefined CT-based imaging and clinical exclusion criteria, 98 patients were included in the final analysis (Fig. 1).

Among the included patients, 54 (55.1%) were male and 44 (44.9%) were female. The median age at transplantation was 59.5 years (interquartile range [IQR] 46.5–65.5; range 18–74).

The most common underlying diagnosis was multiple myeloma (38.8%), followed by non-Hodgkin lymphoma (32.7%), Hodgkin lymphoma (16.3%), and other diagnoses (Table 1).

**Table 1:** Baseline patient characteristics

Variable	Value
Age, years	59.5 (IQR 46.5–65.5)
Male sex	54 (55.1%)
Female sex	44 (44.9%)
Comorbidity Index	1 (IQR 0–3)
Time to early post-transplant CT, days	9.0 (IQR 7.8–13.0)
Time to late post-transplant CT, days†	91 (IQR: 63–176)
Multiple myeloma	38 (38.8%)
Non-Hodgkin lymphoma	32 (32.7%)
Hodgkin lymphoma	16 (16.3%)
CNS lymphoma	8 (8.2%)
Peripheral T-cell lymphoma	3 (3.1%)
Testicular cancer	1 (1.0%)

Values are presented as median (IQR) or number (%). *Abbreviations:* CNS, central nervous system. IQR, interquartile range

^†^Available in patients with late follow-up CT (*n* = 61)

Among patients with early post-transplant CT examinations (*n* = 98), the primary documented indication for CT was fever and/or suspected infection in 43 patients (43.9%), respiratory symptoms and/or hypoxemia in 30 patients (30.6%), and unexplained clinical deterioration in 25 patients (25.5%) (Table 2).

**Table 2:** Clinical indications for early post-transplant CT examinations

Indication	n (%)
Fever and/or suspected infection	43 (43.9)
Respiratory symptoms and/or hypoxemia	30 (30.6)
Unexplained clinical deterioration	25 (25.5)
Total	98 (100)

#### Vertebral attenuation measurements

The mean pre-transplant vertebral trabecular attenuation was 163.0 ± 57.0 HU (range 60–309; *n* = 98).

**Fig. 2:**
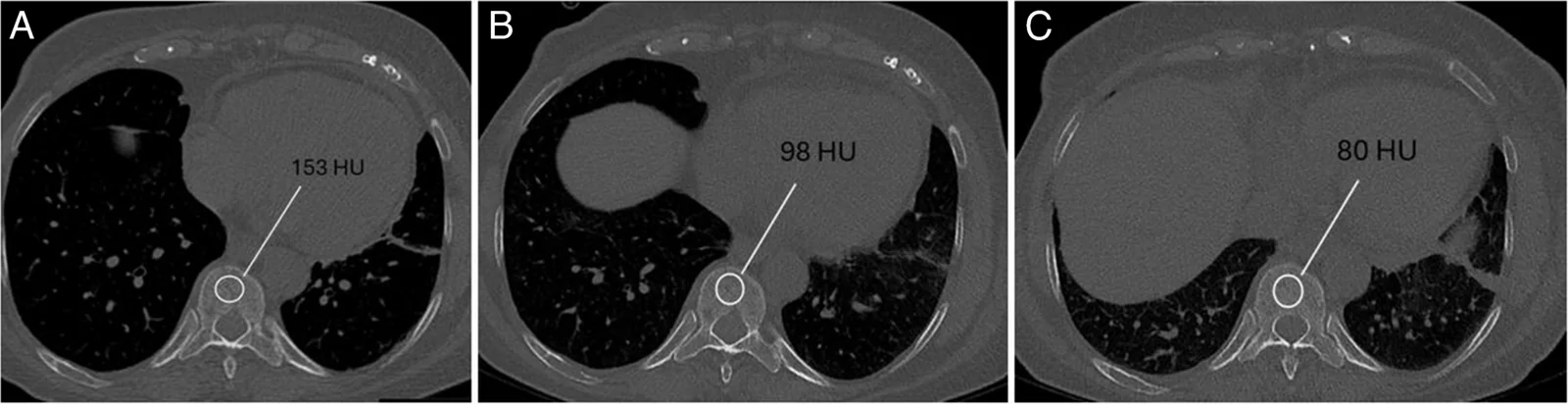
Representative axial non-contrast chest CT images demonstrating vertebral trabecular attenuation changes in a 76-year-old female patient undergoing autologous hematopoietic stem cell transplantation. (**A**) Pre-transplant CT shows normal trabecular attenuation (153 HU). (**B**) Early post-transplant CT at day 13 demonstrates a marked decrease in trabecular attenuation (98 HU). (**C**) Late post-transplant CT at day 134 shows further decline in attenuation (80 HU), consistent with progressive trabecular bone loss. Images are shown at slightly different axial levels because of variations in inspiratory volume and patient positioning between examinations. Vertebral attenuation measurements for study analyses were obtained from standardized ROI measurements at the mid-vertebral level of T10–T12 vertebral bodies, and mean values were used for analysis

Early post-transplant CT examinations were available in 98 patients and were obtained at a median of 9.0 days after transplantation (IQR 7.8–13.0). Mean vertebral attenuation on early CT decreased to 132.7 ± 53.9 HU. Representative examples of vertebral trabecular attenuation before and after transplantation are shown in Fig. 2.

Late follow-up CT examinations were available in 61 patients, with a mean attenuation of 125.3 ± 56.1 HU. The median interval between transplantation and late follow-up CT examination was 91 days (IQR: 63–176 days; mean ± SD: 148.6 ± 150.8 days).

Intraobserver agreement for vertebral trabecular attenuation measurements was excellent, with ICC values of 0.999 for pre-transplant CT, 0.998 for early post-transplant CT, and 0.994 for late follow-up CT.

### Vertebral body height measurements

In the longitudinal follow-up cohort, vertebral body height remained unchanged between the pre-transplant and early post-transplant CT examinations across anterior, middle, posterior, and mean height measurements (all *p* > 0.05). In contrast, significant reductions in vertebral body height were observed at late follow-up compared with both pre-transplant and early post-transplant CT examinations across all measurement levels (all *p* < 0.001) (Table 3). Interobserver agreement was good to excellent across all vertebral levels and time points, with ICC values ranging from 0.798 to 0.999 (Table 4).

**Table 3:** Vertebral body height measurements

**Early follow-up cohort**						
**Measurement**	**Pre-transplant**		**Early post-transplant**		***p* value**	
Anterior height	22.83 ± 1.60		22.86 ± 1.57		0.605	
Middle height	21.08 ± 1.76		21.14 ± 1.83		0.170	
Posterior height	24.93 ± 1.44		24.94 ± 1.45		0.745	
Mean height	22.95 ± 1.49		23.00 ± 1.52		0.351	
**Late follow-up cohort**						
**Measurement (mm)**	**Pre-transplant CT**	**Early post-transplant CT**	**Late follow-up CT**	**P Pre vs Early**	**P Pre vs Late**	**P Early vs Late**
Anterior height	22.92 ± 1.63	22.95 ± 1.61	20.74 ± 1.47	0.605	< 0.001	< 0.001
Middle height	21.10 ± 1.85	21.14 ± 1.97	18.87 ± 1.50	0.093	< 0.001	< 0.001
Posterior height	25.08 ± 1.51	25.08 ± 1.58	21.47 ± 1.60	0.745	< 0.001	< 0.001
Mean height	23.04 ± 1.57	23.07 ± 1.63	20.37 ± 1.37	0.324	< 0.001	< 0.001

Values are presented as mean ± standard deviation. Pairwise comparisons were performed between pre-transplant and early post-transplant CT, pre-transplant and late follow-up CT, and early post-transplant and late follow-up CT using paired t-tests or Wilcoxon signed-rank tests, as appropriate according to the distribution of paired differences. Statistically significant p values are shown in bold

**Table 4:** Interobserver agreement for vertebral body height measurements

Measurement	n	ICC (95% CI)
Pre-transplant anterior height	98	0.910 (0.897–0.953)
Pre-transplant middle height	98	0.974 (0.962–0.983)
Pre-transplant posterior height	98	0.984 (0.976–0.989)
Early post-transplant anterior height	98	0.994 (0.990–0.996)
Early post-transplant middle height	98	0.999 (0.999–1.000)
Early post-transplant posterior height	98	0.992 (0.988–0.994)
Late follow-up anterior height	61	0.940 (0.907–0.965)
Late follow-up middle height	61	0.798 (0.680–0.872)
Late follow-up posterior height	61	0.918 (0.865–0.949)

Interobserver agreement was assessed using the intraclass correlation coefficient (ICC). Values are presented as ICC with 95% confidence intervals (CI). According to commonly accepted criteria, ICC values < 0.50 indicate poor, 0.50–0.75 moderate, 0.75–0.90 good, and > 0.90 excellent agreement

#### Early attenuation changes

Vertebral trabecular attenuation decreased significantly between pre-transplant and early post-transplant CT (163.0 ± 57.0 HU vs 132.7 ± 53.9 HU; mean ΔHU − 30.3 ± 22.4; *p* < 0.001, Wilcoxon signed-rank test).

### Longitudinal attenuation changes

Among patients with available late follow-up CT (*n* = 61), vertebral attenuation remained significantly lower than baseline (157.3 ± 58.7 HU vs 125.3 ± 56.1 HU; mean ΔHU − 32.0 ± 30.1; *p* < 0.001). Attenuation measurements in the longitudinal follow-up cohort are summarized in Table 5. No significant difference was observed between early and late attenuation values (*p* = 0.678), suggesting that attenuation decline occurred predominantly during the early post-transplant period and remained relatively stable thereafter.

**Table 5:** Vertebral trabecular attenuation measurements in the longitudinal follow-up cohort (*n* = 61)

Time point	Mean attenuation (HU)
Pre-transplant CT	157.3 ± 58.7
Early post-transplant CT	129.6 ± 55.2
Late follow-up CT	125.3 ± 56.1

Values are presented as mean ± SD. Vertebral attenuation was significantly lower at both early and late follow-up compared with baseline (both *p* < 0.001), whereas no significant difference was observed between early and late follow-up (*p* = 0.678)

**Fig. 3:**
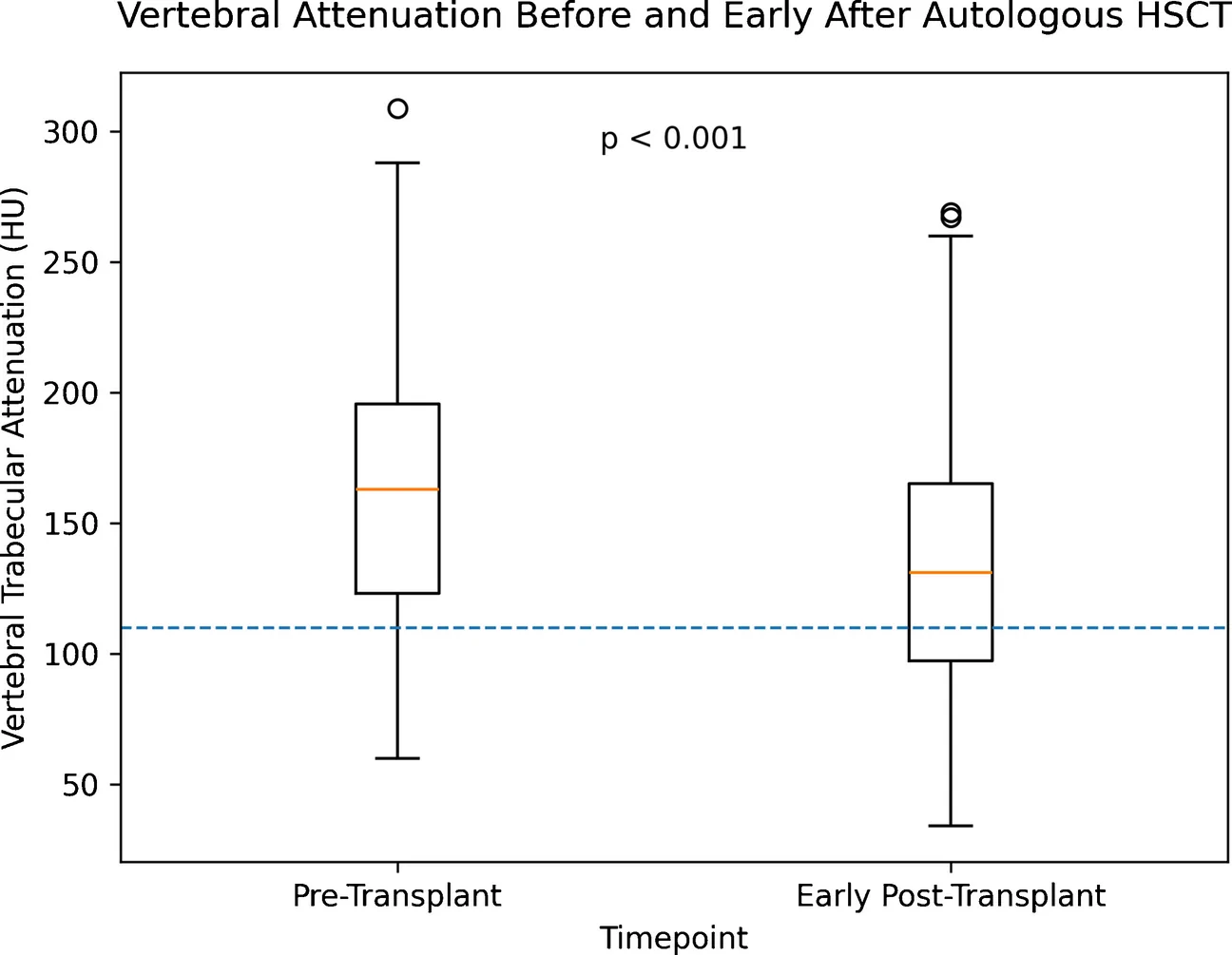
Vertebral trabecular attenuation before and early after autologous HSCT. Boxplots illustrate attenuation values at baseline and in the early post-transplant period in the full cohort (*n* = 98). Vertebral attenuation decreased significantly in the early post-transplant period (*p* < 0.001). The dashed horizontal line indicates the commonly used osteoporosis threshold (110 HU)

Individual patient trajectories and cohort mean attenuation values are shown in Fig. 3.

### Distribution of attenuation changes

**Fig. 4:**
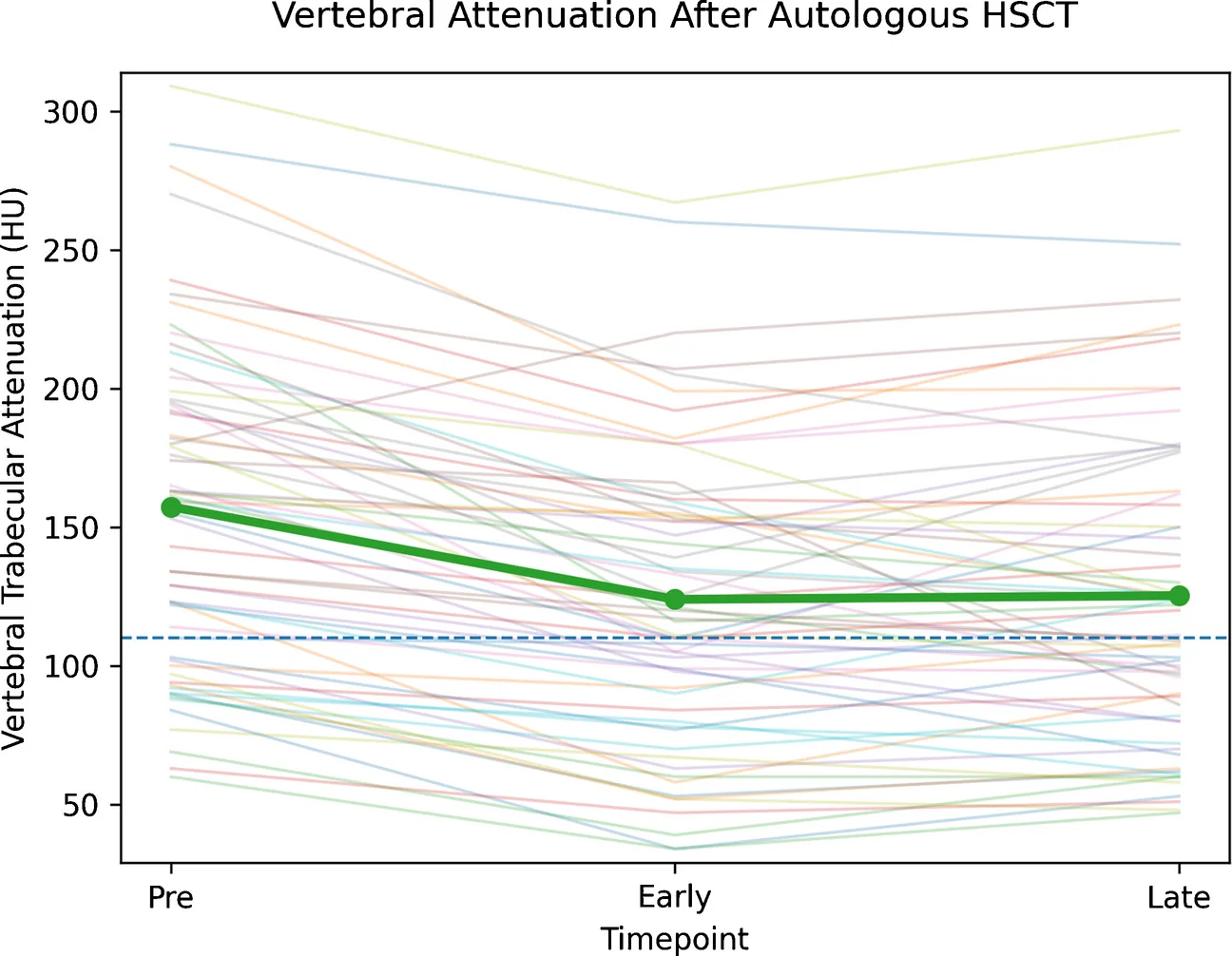
Longitudinal changes in vertebral trabecular attenuation after autologous HSCT. Individual patient trajectories are shown for patients with available late follow-up CT (*n* = 61). Each thin line represents an individual patient, and the thick line indicates the cohort mean. Vertebral attenuation declines in all but one patient early in the post-transplant period and remains reduced at follow-up in the majority of patients

The distribution of attenuation changes relative to baseline is shown in Fig. 4. Both early and late post-transplant measurements demonstrated predominantly negative ΔHU values, indicating a consistent decline in vertebral trabecular attenuation following transplantation.

### Linear regression analysis

In linear regression analysis, greater attenuation decline was associated with older age (β − 0.49 HU/year, *p* = 0.004) and higher baseline vertebral attenuation (β − 0.17, *p* < 0.001), whereas sex was not significantly associated with attenuation change (*p* = 0.81).

### Exploratory attenuation threshold analysis

**Fig. 5:**
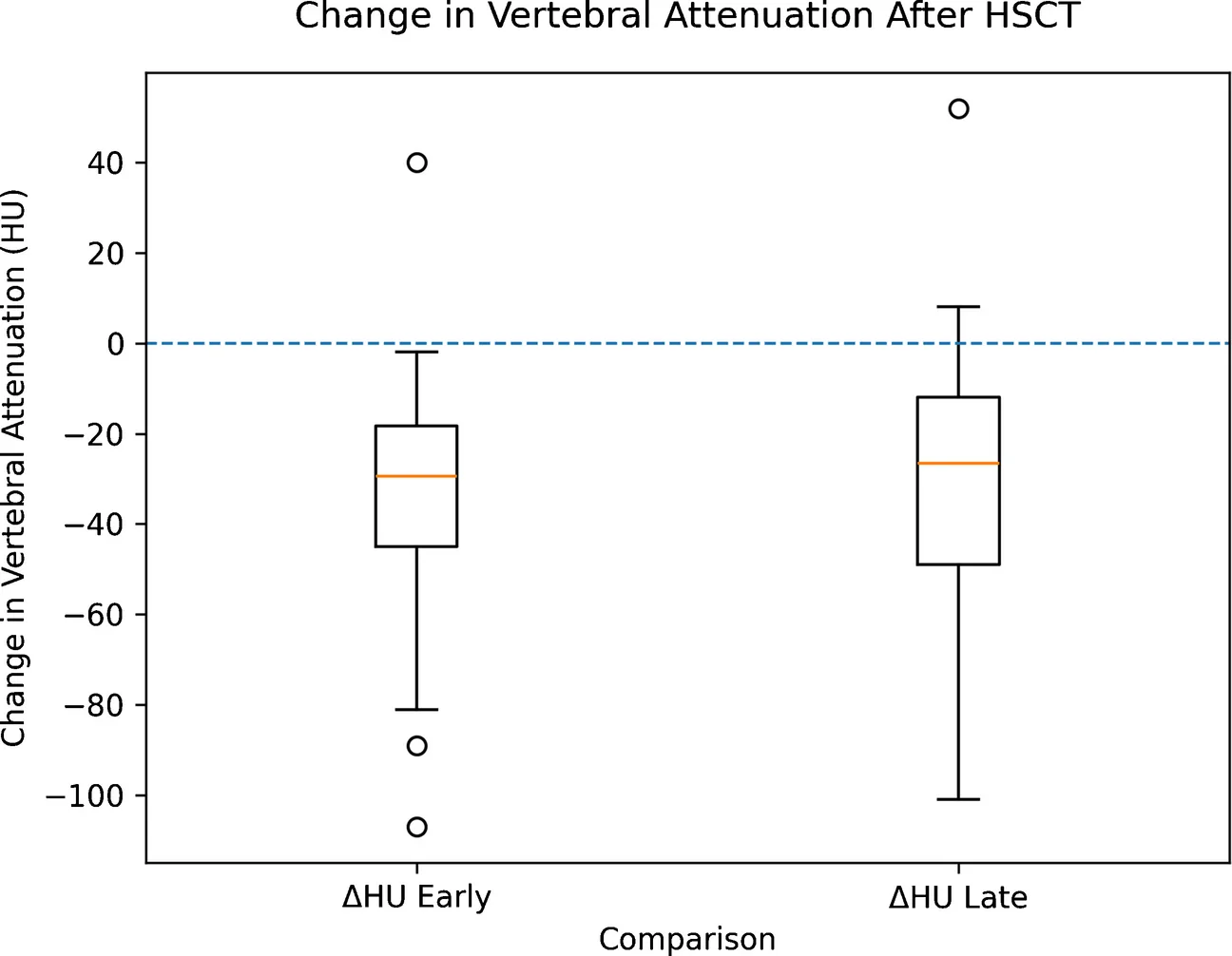
Distribution of changes in vertebral trabecular attenuation following transplantation. Boxplots show changes in attenuation (ΔHU) between baseline and early post-transplant CT (ΔHU Early) and between baseline and late follow-up CT (ΔHU Late). Negative values indicate attenuation decline. The dashed horizontal line represents no change (ΔHU = 0)

Changes in the prevalence of osteoporosis-range attenuation are illustrated in Fig. 5.

The proportion of vertebral attenuation values within the osteoporosis range (≤ 110 HU) increased from 20.4% at baseline to 39.8% in the early post-transplant period in the full cohort.

Among patients with available late follow-up imaging (*n* = 61), the proportion of attenuation values ≤ 110 HU increased from 27.4% at baseline to 45.2% early after transplantation, and remained elevated at 48.4% at late follow-up.

## Discussion

### Principal findings

In this study, vertebral trabecular attenuation measured on non-enhanced chest CT demonstrated a marked decline within the early post-transplant period following autologous HSCT. The reduction in attenuation was substantial, with a mean decrease of approximately 30 HU within the first month after transplantation. Importantly, attenuation values remained persistently reduced on subsequent follow-up imaging, with no significant recovery observed between early and late post-transplant CT examinations. These findings suggest that the majorityof skeletal attenuation loss occurs during the immediate peri-transplant period.

Consequent to the attenuation decline, a substantial proportion of vertebral attenuation values fell within the osteoporosis range (≤ 110 HU). In the full cohort, the proportion of patients with attenuation values within this range increased from 20% at baseline to 40% in the early post-transplant period, indicating a rapid shift toward skeletal vulnerability shortly after transplantation.

### Relationship to previous literature

Bone loss and fracture risk after transplantation are well established in the literature. Large population-based analyses have demonstrated significantly increased risks of osteoporosis and fragility fractures in transplant recipients compared with the general population [1, 12]. Early studies in lung transplantation similarly reported a high incidence of fractures during the first year after transplantation [2, 13].

Most prior studies have evaluated skeletal health using dual-energy X-ray absorptiometry (DXA) performed months after transplantation [3, 4, 14, 15]. However, there is a lack of studies examining very early skeletal changes during the immediate peri-transplant period.

Opportunistic osteoporosis assessment using routine CT has gained increasing interest. Vertebral trabecular attenuation measured on CT has been shown to correlate with bone mineral density and fracture risk. Large cohort studies have established reference values for vertebral attenuation and demonstrated the feasibility of CT-based osteoporosis screening without additional imaging or radiation exposure [7, 16–18]. Furthermore, CT-derived vertebral attenuation has been shown to outperform conventional fracture risk prediction tools in certain populations [8, 19].

Our findings extend this literature by demonstrating that measurable attenuation decline occurs very early after HSCT, potentially preceding the time frame in which DXA is typically performed.

### Possible mechanisms

The rapid attenuation decline observed in this study likely reflects multiple interacting mechanisms. The early post-transplant period is characterized by intense systemic physiological stress, including inflammatory activation, metabolic disturbances, medication exposure, and reduced mobility.

Glucocorticoids and immunosuppressive therapies are well-recognized contributors to transplant-associated bone loss. The former promote osteoclast activity while inhibiting osteoblast function, leading to accelerated bone resorption [3, 20, 21]. In addition, persistent disturbances in mineral metabolism may occur even after transplantation, further contributing to skeletal fragility [5, 22].

Importantly, the magnitude and rapidity of attenuation decline observed in this study suggest that early CT attenuation changes may reflect not only alterations in trabecular bone structure but also changes in marrow composition or fluid content during the acute post-transplant period. Higher baseline attenuation was associated with greater early attenuation decline, which may support the possibility of treatment-related marrow contribution to attenuation changes. Nevertheless, the persistent attenuation reduction observed on follow-up CT, together with progressive decreases in vertebral body height, suggests that structural skeletal alterations may also contribute to these findings.

Vertebral body height findings provide additional insight into the temporal evolution of skeletal changes following autologous HSCT. Despite the marked decline in vertebral trabecular attenuation during the early post-transplant period, vertebral body height remained unchanged across anterior, middle, posterior, and mean measurements. Significant reductions in vertebral body height were observed only at late follow-up, suggesting that measurable morphologic changes develop more gradually than alterations in trabecular attenuation. This temporal dissociation supports the concept that CT-derived trabecular attenuation may represent an earlier imaging marker of skeletal deterioration than vertebral body height measurements. Although vertebral height assessment is inherently more susceptible to technical and positional variability than attenuation measurements, the good-to-excellent interobserver agreement observed in the present study supports the reproducibility of this approach. Nevertheless, vertebral height findings should be interpreted with appropriate caution and regarded as supportive rather than definitive evidence of structural skeletal alteration.

### Clinical implications

Our findings highlight the potential value of opportunistic CT-based bone assessment in transplant recipients. Chest CT is frequently performed in patients undergoing HSCT for infection surveillance or evaluation of respiratory complications. Vertebral trabecular attenuation can therefore be assessed without additional imaging, radiation exposure, or cost.

Early identification of attenuation decline may allow clinicians to recognize patients at increased skeletal risk and consider earlier bone health evaluation or preventive strategies. Opportunistic CT-based screening approaches may therefore complement traditional DXA-based assessment and provide clinically relevant information during the early post-transplant period. However, opportunistic CT-based attenuation assessment should be considered complementary to, rather than a replacement for, standard DXA-based osteoporosis evaluation.

### Limitations

Several limitations should be acknowledged. First, this was a retrospective single-center study, which may limit the generalizability of the findings. Second, post-transplant chest CT examinations were performed for clinical indications rather than according to a predefined imaging protocol. Consequently, only patients who underwent clinically indicated follow-up CT examinations were included, and these individuals may have represented a clinically more vulnerable subgroup than the overall autologous HSCT population, introducing potential selection bias. Third, although CT acquisition parameters were standardized as much as possible by including only non-contrast examinations acquired at 120 kVp using consistent reconstruction algorithms, attenuation measurements may still have been influenced by residual technical variability. Fourth, although glucocorticoid exposure as part of the conditioning regimen was standardized and no patient received post-transplant immunosuppressive therapy, detailed information regarding cumulative exposure to other medications affecting bone metabolism (e.g., antiresorptive therapy) was not consistently available. Therefore, the independent contribution of these factors to the observed attenuation changes could not be evaluated. Fifth, vertebral body height measurements were not available for all patients because late follow-up CT examinations were obtained only when clinically indicated. However, interobserver reproducibility was assessed using independent blinded measurements performed by a second radiologist and demonstrated good-to-excellent agreement across all vertebral levels and time points. Finally, vertebral trabecular attenuation represents an opportunistic imaging biomarker rather than a direct measurement of bone mineral density and was not validated against contemporaneous DXA measurements, quantitative CT, bone turnover biomarkers, or incident fragility fractures. Accordingly, prospective longitudinal studies incorporating these complementary assessments are needed to determine the clinical significance of the early attenuation decline observed after autologous HSCT.

## Conclusion

Vertebral trabecular attenuation measured on routine chest CT declined rapidly within the first month after autologous HSCT, whereas vertebral body height remained unchanged during the early post-transplant period and decreased only at later follow-up. These findings suggest that opportunistic CT-derived trabecular attenuation may serve as an earlier imaging marker of skeletal deterioration than morphologic vertebral changes and may help identify patients who could benefit from earlier bone health assessment.

## Data Availability

The datasets generated and analysed during the current study are available from the corresponding author on reasonable request.

## Abbreviations

CNS: Central nervous system
CT: Computed tomography
DXA: Dual-energy X-ray absorptiometry
HSCT: Hematopoietic stem cell transplantation
HU: Hounsfield units
ICC: Intraclass correlation coefficient
IQR: Interquartile range
ROI: Region of interest
SD: Standard deviation
